# Population-Based Estimates of Chronic Conditions Affecting Risk for Complications from Coronavirus Disease, United States

**DOI:** 10.3201/eid2608.200679

**Published:** 2020-08

**Authors:** Mary L. Adams, David L. Katz, Joseph Grandpre

**Affiliations:** On Target Health Data LLC, Suffield, Connecticut, USA (M.L. Adams);; True Health Initiative, Derby, Connecticut, USA (D.L. Katz);; Wyoming Department of Health, Cheyenne, Wyoming, USA; (J. Grandpre)

**Keywords:** population-based estimates, chronic conditions, risk factors, complications, coronavirus disease, COVID-19, severe acute respiratory syndrome coronavirus 2, SARS-CoV-2, infection, viruses, respiratory infections, zoonoses, United States

## Abstract

We estimated that 45.4% of US adults are at increased risk for complications from coronavirus disease because of cardiovascular disease, diabetes, respiratory disease, hypertension, or cancer. Rates increased by age, from 19.8% for persons 18–29 years of age to 80.7% for persons >80 years of age, and varied by state, race/ethnicity, health insurance status, and employment.

Data for China indicate that 81% of coronavirus disease (COVID-19) patients had mild cases, 14% had severe cases, and 5% had critical cases (*1**,**2*). The overall case-fatality rate (CFR) in China was 3.8% (*3*), but CFRs were higher for adults with chronic conditions of cardiovascular disease (CVD; CFR 13.2%), diabetes (9.2%), chronic respiratory disease (8.0%), hypertension (8.4%), and cancer (7.6%), compared with 1.4% for patients with none of these conditions (*3*). Our objective for this study was to use population-based US data to estimate the fraction of adults in the community who might be at increased risk for complications from COVID-19 because they reported any of the chronic conditions with a high CFR in China.

## The Study

We used publicly available 2017 Behavioral Risk Factor Surveillance System (BRFSS) data (*4*) from telephone surveys of 444,649 randomly selected adults >18 years of age in the 50 states and the District of Columbia (DC). Because the BRFSS includes only noninstitutionalized adults, residents of nursing homes and assisted living facilities are among those not surveyed. We chose to use 2017 data to include hypertension, which was not addressed in 2018. Data were adjusted for the probability of selection and weighted it to be representative of the adult population in each state by age, sex, race/ethnicity, marital status, education, home ownership, and telephone type. Weights and stratum variables needed for analysis were included.

We did not age-adjust results in order to reflect the age distribution of each state rather than a standard population. The median response rate for cell phone and landline surveys combined was 47.2%, ranging from 33.9% in California to 61.1% in Utah (*5*). Reliability and validity of the BRFSS have been found to be moderate to high for many survey measures, in particular those used here, which can be checked versus medical records (*6*).

The key variable was a composite measure including adults reporting that they were ever told they had CVD (heart attack, angina, coronary heart disease, or stroke), diabetes, asthma, chronic obstructive pulmonary disease, hypertension, or cancer other than skin. We counted the number of chronic conditions for each respondent and adults who reported >1 condition and were considered to be at heightened risk for complications from COVID-19. A secondary measure was receipt of a seasonal influenza vaccination in the past year as a rough estimate of potential demand for a COVID-19 vaccine when available.

Demographic measures included age group (18–29, 30–39, 40–49, 50–59, 60–69, 70–79, and >80 years of age; we created these measures by combining 5-year age groups provided in the dataset); self-reported race/ethnicity (non-Hispanic White, Black or African American, Hispanic of any race, American Indian/Alaska native, Asian/Pacific Islander, and other); health insurance coverage (any kind of healthcare coverage, including health insurance, prepaid plans such as health maintenance organizations, or government plans such as Medicare, or Indian Health Service); employment status (employed or self-employed, out of work, homemaker, student, retired, or unable to work); and state of residence, which included DC.

We used Stata version 14.1 (StataCorp LP, https://www.stata.com) for analysis to account for the complex sample design of the BRFSS. We report point estimates and 95% CIs or population estimates by using weights, stratum, and primary sampling unit variables supplied in the dataset (*4*). Missing values were excluded from analysis.

Among 444,649 survey respondents, 48.7% were male, 13.9% were >70 years of age, 63.3% were white, 18.2% were retired, and 12.1% were uninsured. We obtained similar results for the study sample when 11,508 records that had missing values were removed. Overall, 45.4% (95% CI 45.1%–45.7%) of respondents fit the description of being at heightened risk for complications from COVID-19.

Among all adults, 26.7% (95% CI 26.5%–27.0%) reported 1 chronic condition, 12.0% (95% CI 11.8%–12.2%) reported 2 chronic conditions, 4.7% (95% CI 4.6%–4.8%) reported 3 chronic conditions, and 2.0% (95% CI 1.9%–2.1%) reported >4 chronic conditions. Prevalence rates of separate chronic conditions were 8.5% for CVD, 6.6% for chronic obstructive pulmonary disease, 9.1% for asthma (and still have it), 10.8% for diabetes, 32.4% for hypertension, and 6.8% for cancer. Although the percentage of adults with any of the chronic conditions increased with age (Table 1), more than half (53.4%) of the total were 18–59 years of age. Rates also varied by state, race/ethnicity, insurance status, and employment, but not by sex (Table 1).

**Table 1:** Demographics of adults with any of 6 chronic conditions increasing risk for coronavirus disease complications, 2017 Behavioral Risk Factor Surveillance System*

Characteristic	% With >1 condition (95% CI)	Sample size
Total	45.4 (45.1–45.7)	433,141
Sex		
M	45.4 (44.9–45.9)	191,193
F	45.4 (45.0–45.9)	241,695
Age, y		
18–29	19.8 (19.1–20.4)	46,660
30–39	26.8 (26.0–27.5)	49,475
40–49	38.1 (37.3–39.0)	53,609
50–59	55.1 (54.3–55.9)	79,550
60–69	68.0 (67.3–68.6)	96,663
70–79	79.5 (78.7–80.2)	67,733
>80	80.7 (79.5–81.8)	33,757
Race/ethnicity		
White, non-Hispanic	48.0 (47.7–48.4)	329,193
Black	52.1 (51.1–53.1)	35,087
Hispanic	35.5 (34.5–36.5)	31,624
American Indian/Alaska Native	55.5 (52.9–58.1)	8,082
Asian/Pacific Islander	27.8 (25.9–29.7)	10,117
Other	46.5 (44.5–48.4)	10,854
Employment		
Employed/self-employed	35.4 (35.0–35.8)	217,975
Out of work	47.0 (45.5–48.5)	18,644
Homemaker	39.6 (38.2–41.0)	22,790
Student	18.1 (16.9–19.4)	11,634
Retired	75.7 (75.1–76.3)	128,162
Unable to work	79.3 (78.3–80.3)	30,510
Insurance status		
Insured	47.1 (46.8–47.5)	397,495
Uninsured	33.4 (32.4–34.4)	34,052
State		
AL	54.2 (52.6–55.9)	6,565
AK	43.6 (40.7–46.5)	3,124
AZ	44.6 (43.6–45.6)	15,086
AR	53.3 (50.7–55.8)	5,126
CA	41.0 (39.6–42.4)	9,149
CO	40.0 (38.8–41.2)	9,486
CT	45.0 (43.7–46.3)	10,298
DE	48.7 (46.5–50.9)	4,015
DC	38.3 (36.4–40.1)	4,400
FL	46.9 (45.4–48.5)	21,442
GA	45.6 (44.0–47.2)	5,895
HI	44.0 (42.5–45.5)	7,645
ID	42.6 (40.7–44.5)	4,791
IL	44.9 (43.3–46.6)	5,498
IN	48.7 (47.5–49.8)	13,489
IA	44.9 (43.6–46.2)	7,553
KS	45.9 (45.1–46.8)	21,233
KY	53.2 (51.5–55.0)	8,391
LA	51.6 (49.7–53.5)	4,680
ME	49.9 (48.3–51.5)	9,513
MD	44.9 (43.5–46.2)	13,179
MA	43.4 (41.5–45.4)	6,670
MI	49.4 (48.2–50.6)	10,584
MN	38.8 (37.9–39.7)	16,714
MS	51.9 (49.8–54.0)	4,937
MO	46.2 (44.6–47.8)	7,394
MT	44.0 (42.3–45.8)	5,793
NE	43.4 (42.2–44.6)	15,039
NV	46.1 (43.7–48.5)	3,683
NH	45.9 (44.0–47.8)	5,605
NJ	45.6 (44.0–47.1)	11,399
NM	45.3 (43.5–47.0)	6,376
NY	42.2 (41.0–43.4)	11,951
NC	47.5 (45.6–49.3)	4,800
ND	42.0 (40.5–43.6)	6,862
OH	48.3 (46.9–49.6)	11,975
OK	50.7 (49.0–52.3)	6,369
OR	44.7 (43.1–46.3)	)5,186
PA	47.8 (46.2–49.5)	6,405
RI	47.8 (45.9–49.8)	5,444
SC	49.9 (48.6–51.3)	10,953
SD	43.3 (41.2–45.5)	6,844
TN	51.1 (49.3–53.0)	5,679
TX	43.7 (41.8–45.5)	11,941
UT	37.3 (36.2–38.5)	9,994
VT	45.8 (44.1–47.5)	6,346
VA	45.2 (43.8–46.6)	9,394
WA	44.0 (42.9–45.1)	12,822
WV	58.7 (57.0–60.4)	5,332
WI	43.9 (42.1–45.7)	5,716
WY	44.0 (42.2–45.9)	4,376

*Chronic conditions: cardiovascular disease, diabetes, chronic obstructive pulmonary disease, asthma, hypertension, or cancer other than skin.

State results obtained directly from Stata (Table 2) list the number of adults in each state at increased risk for complications and the percentage that number represents among all states. Results for reporting a seasonal influenza vaccination in the past year were 40.3% (95% CI 40.0%–40.6%) for all adults, including 33.7% (95% CI 33.3%–34.2%) for adults who had none of the chronic conditions and 48.0% (95% CI 47.5%–48.5%) for persons who had any of the 6 chronic conditions.

**Table 2:** Number of adults with any of 6 chronic conditions increasing risk for coronavirus disease complications and percentage of total in each state, 2017 Behavioral Risk Factor Surveillance System, United States*

State	No. adults at risk	% Adults at risk†
AL	1,997,864	1.78
AK	237,208	0.21
AZ	2,351,799	2.10
AR	1,181,105	1.06
CA	12,240,142	10.93
CO	1,701,776	1.52
CT	1,239,597	1.11
DE	357,530	0.32
DC	213,357	0.19
FL	7,696,749	6.88
GA	3,541,358	3.16
HI	486,156	0.43
ID	534,533	0.48
IL	4,404,556	3.93
IN	2,428,188	2.17
IA	1,067,133	0.95
KS	983,323	0.88
KY	1,789,444	1.60
LA	1,806,330	1.61
ME	530,809	0.47
MD	2,054,758	1.84
MA	2,302,809	2.06
MI	3,749,235	3.35
MN	1,625,778	1.45
MS	1,150,036	1.03
MO	2,137,650	1.91
MT	356,113	0.32
NE	616,905	0.55
NV	1,048,591	0.94
NH	485,340	0.43
NJ	3,106,880	2.78
NM	701,585	0.63
NY	6,419,321	5.73
NC	3,713,582	3.32
ND	243,096	0.22
OH	4,268,748	3.81
OK	1,461,941	1.31
OR	1,418,689	1.27
PA	4,738,414	4.23
RI	393,069	0.35
SC	1,912,134	1.71
SD	281,110	0.25
TN	2,610,800	2.33
TX	8,977,387	8.02
UT	796,721	0.71
VT	226,397	0.20
VA	2,921,171	2.61
WA	2,464,452	2.20
WV	827,193	0.74
WI	1,949,872	1.74
WY	194,544	0.17
Total	111,943,278	100

*Chronic conditions: cardiovascular disease, diabetes, chronic obstructive pulmonary disease, asthma, hypertension, or cancer other than skin. †Values were obtained directly from state and will not agree with calculations made from raw data.

## Conclusions

We estimated that 45.4% of US adults, with a wide range across age groups and states, might be at increased risks for complications from COVID-19 because of existing chronic conditions. The 18.7% who reported >2 chronic conditions might be at even greater risk on the basis of other studies (*7*). Although complication rates increased with increasing age, 53% of those at greater risk for complications were <60 years of age. Preliminary data suggest that some of these same chronic conditions increase risk for COVID-19 complications in the United States (*8*). Another BRFSS study that omitted hypertension and used a different definition of risk estimated that 37.6% of US adults were at risk for complications (*9*).

The list of chronic conditions used in our analysis is similar to groups at increased risk for seasonal influenza complications (*10*), except that the influenza group includes obese adults. Obesity is much lower in China than in the United States (*11*), which might account for that difference. Both lists include persons with chronic diseases for which behavioral risk factors have been well identified (*12*,*13*). In particular, the 7 risk factors of smoking, sedentary lifestyle, obesity, diabetes, hypertension, high cholesterol, and inadequate fruit and vegetable consumption together contributed to an average of 41.4% of the burden of 5 of the 6 chronic conditions used in our study (all except cancer); obesity and smoking contributed the most to the burden (*12*). Results showing seasonal influenza vaccination rates <50% are concerning. Although a vaccine specific for this coronavirus is currently unavailable, results for seasonal influenza vaccination suggest that it might not be widely used.

Our study does not address possible differences in contracting the disease, only the risk for development of complications among persons who have COVID-19 on the basis of results for China (*1**–**3*). Because we surveyed only noninstitutionalized adults, we excluded 1.3 million adults in nursing homes (*14*), which almost certainly underestimates risk. Data are self-reported, and reliability and validity can vary for different measures tested (*6*). However, as long as a respondent was told they had a chronic condition, validity was high. Age groups used for analysis did not match those used for weighting data, but that limitation should have a minimal effect on results. Low response rates could introduce bias but, as noted, validity appears high for the measures used in this study.

We estimated that 45.4% of US adults are potentially at increased risk for complications from COVID-19 because of chronic conditions that are, in turn, associated with common modifiable risk factors. Such estimates will vary depending on exact criteria used and the prevalence of the risk factors associated with the chronic conditions, along with age, state of residence, and other demographic factors.
